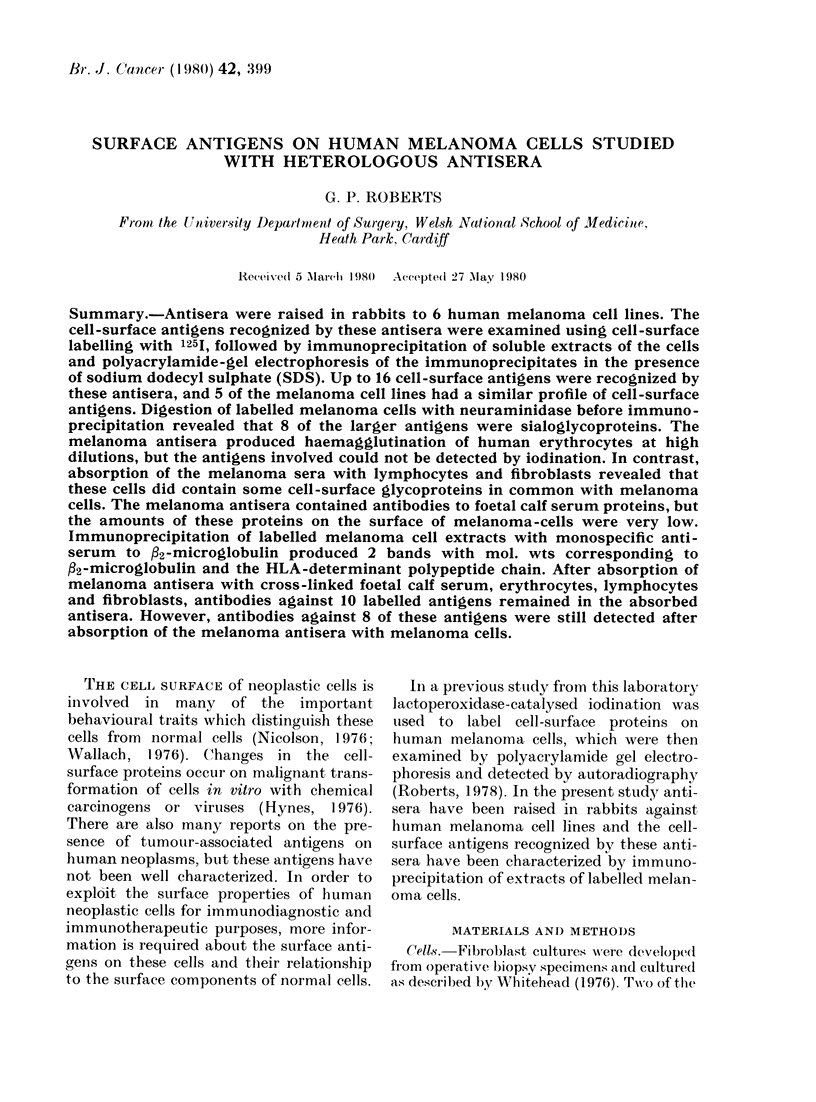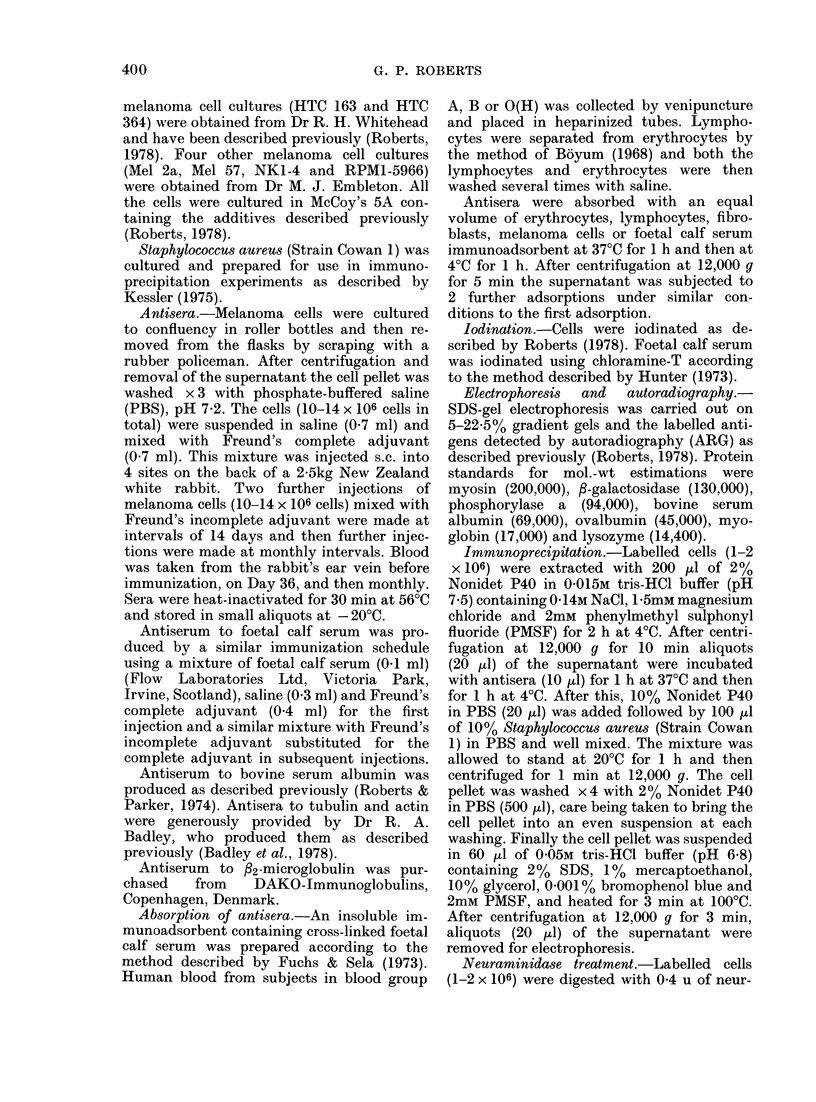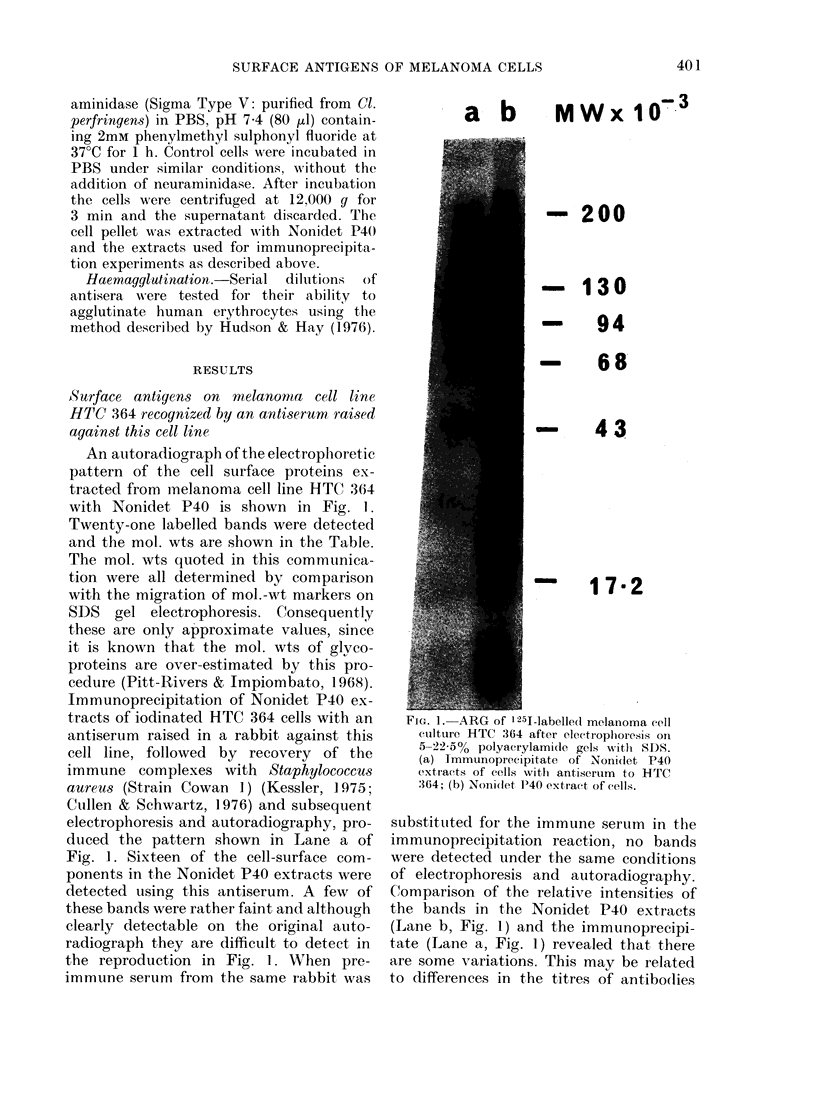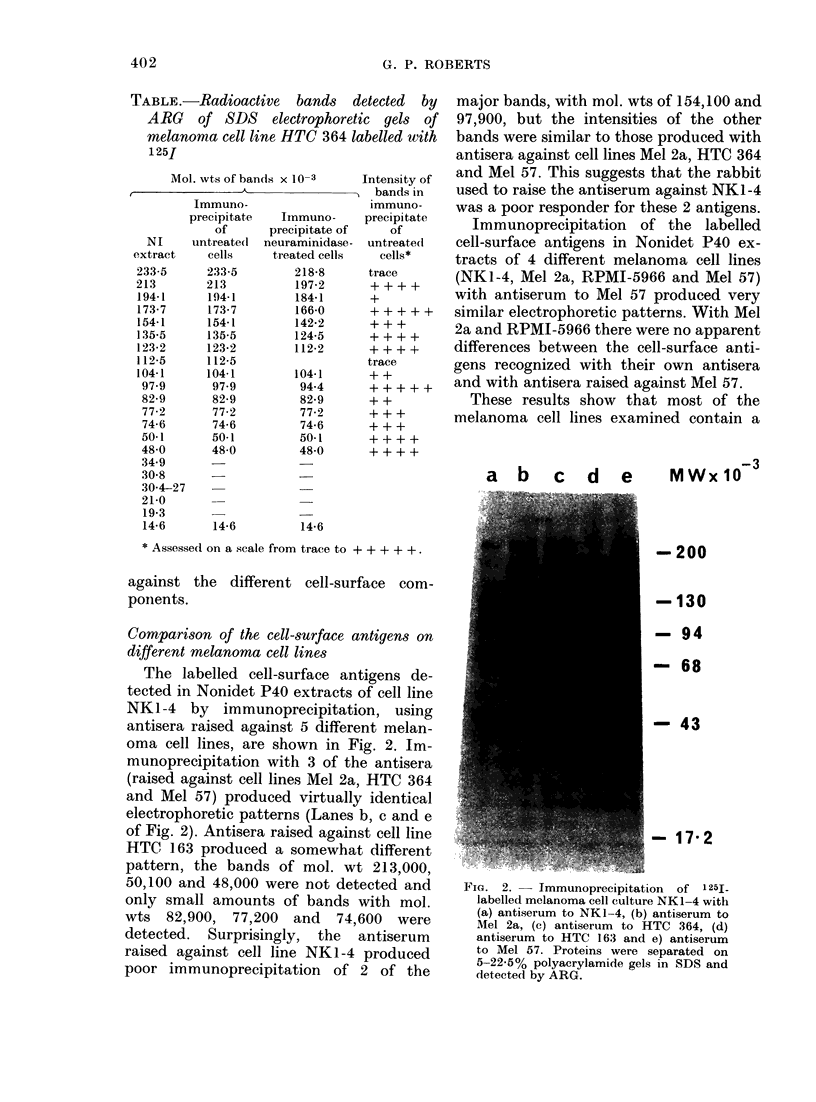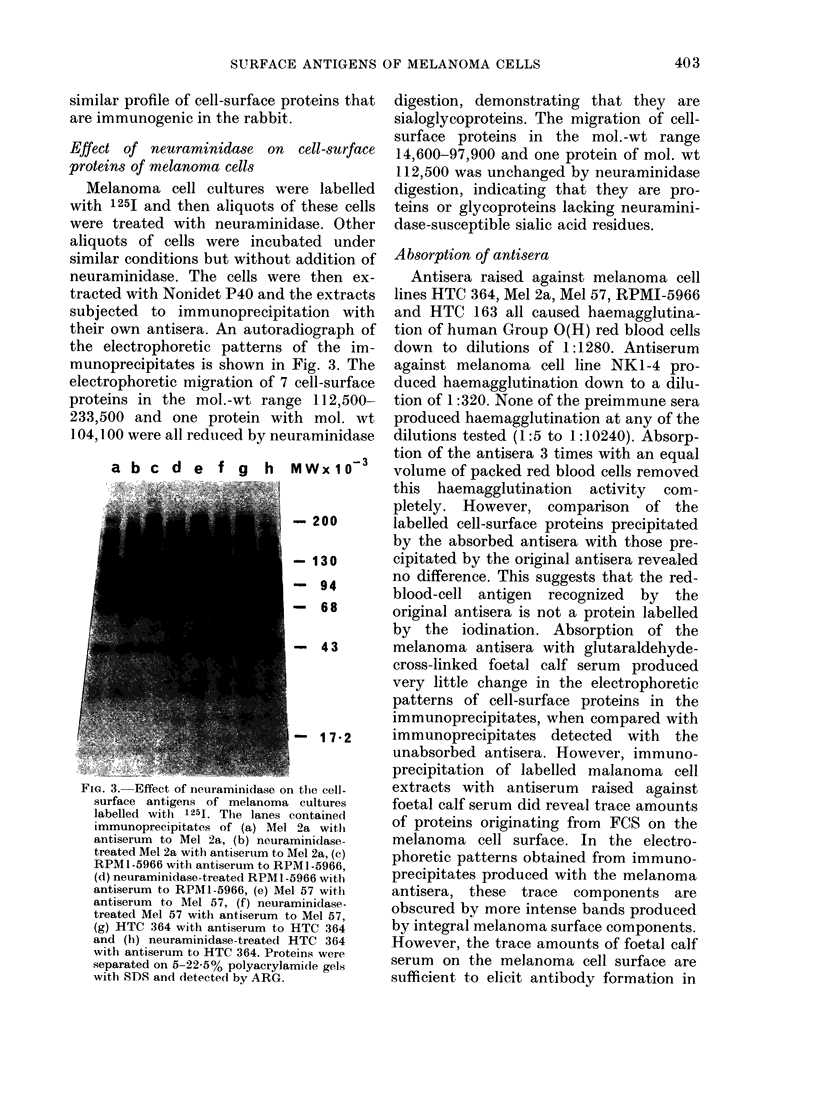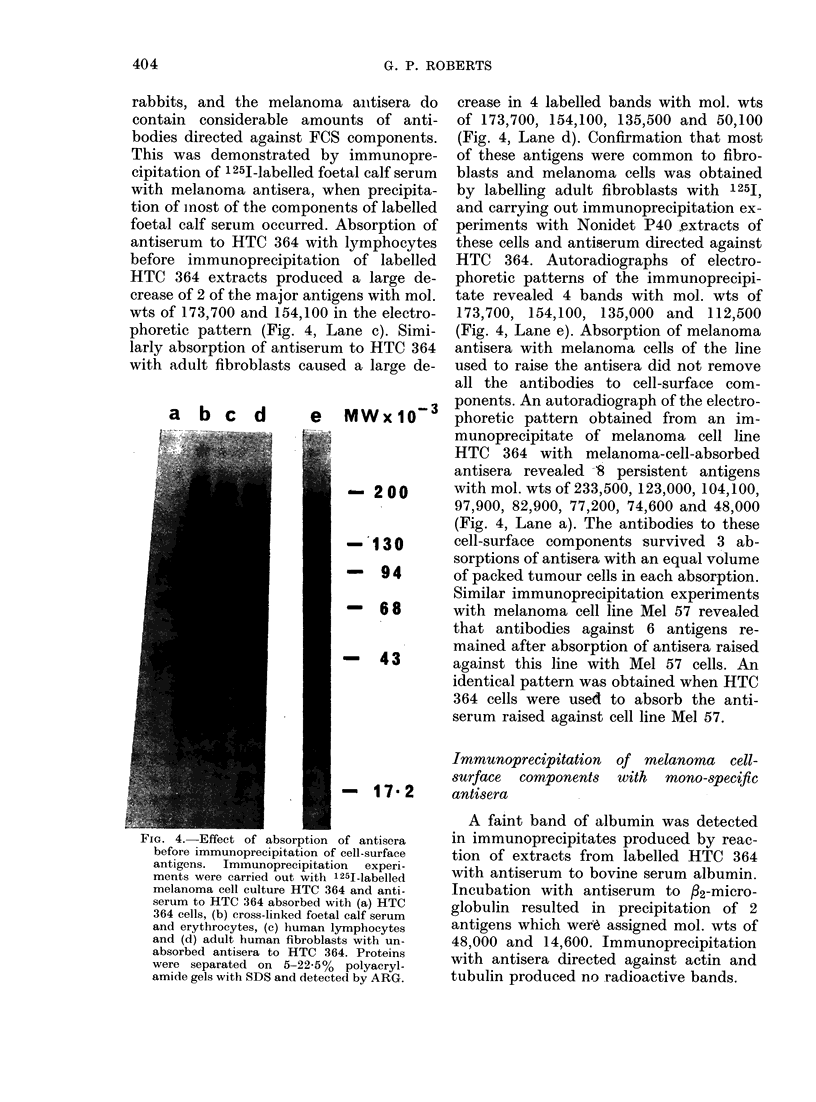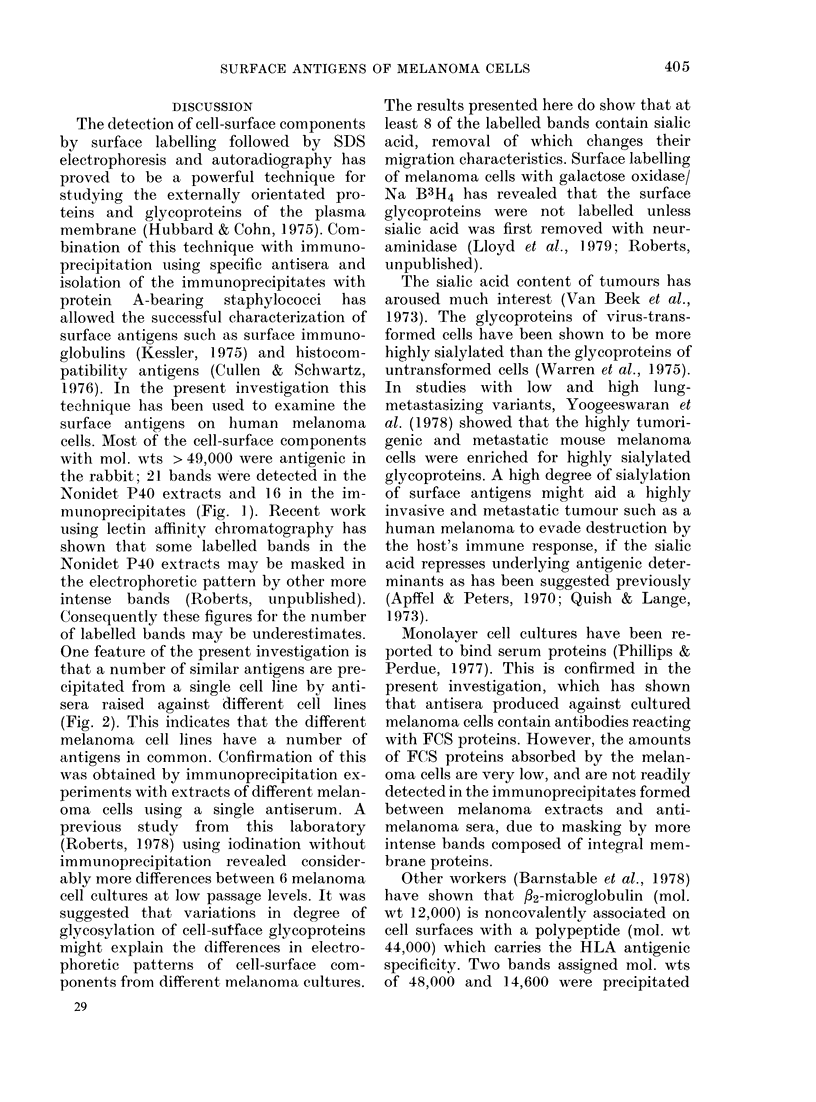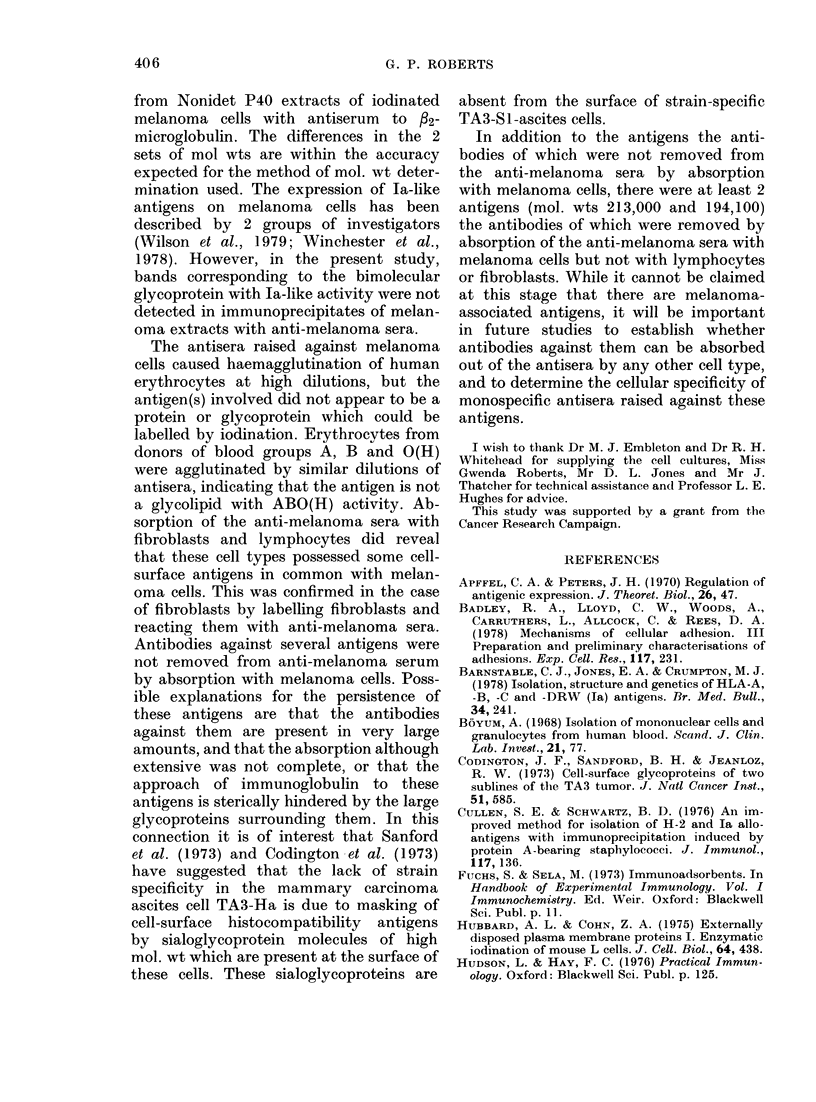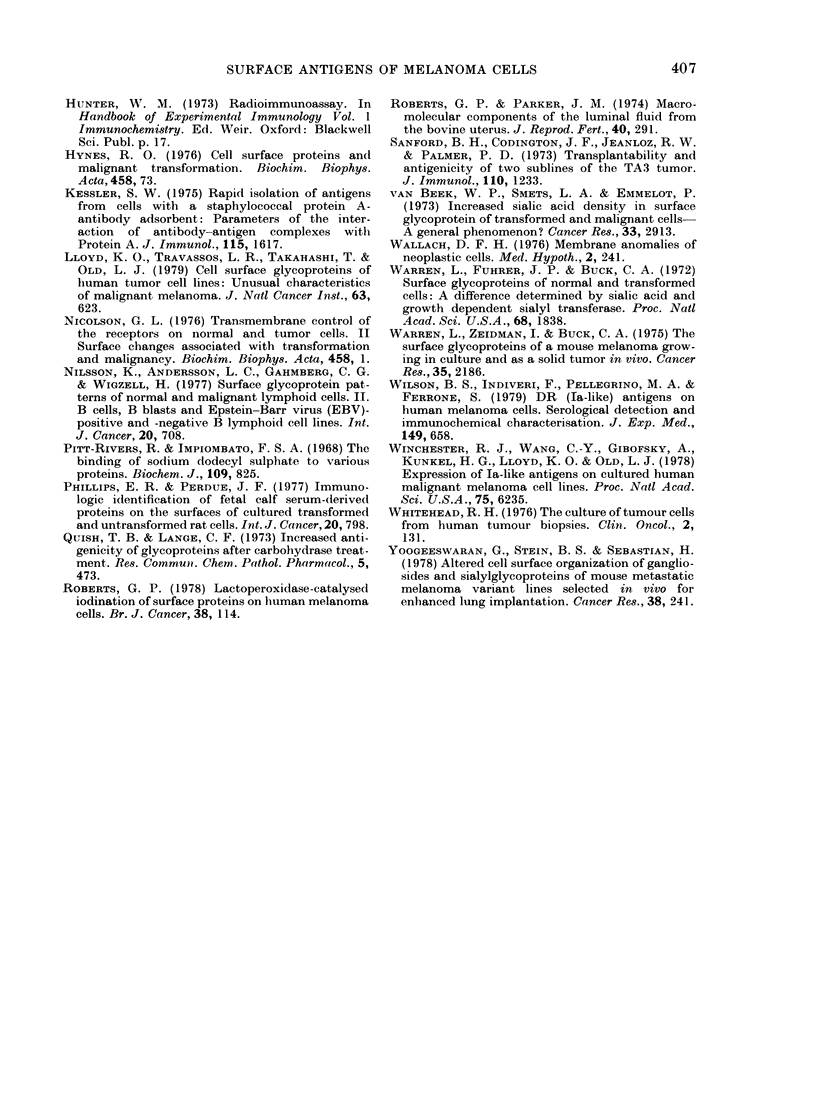# Surface antigens on human melanoma cells studied with heterologous antisera

**DOI:** 10.1038/bjc.1980.251

**Published:** 1980-09

**Authors:** G. P. Roberts

## Abstract

**Images:**


*Br. J. Cancer* (1980) **42**, 399

### SURFACE ANTIGENS ON HUMAN MELANOMA CELLS STUDIED WITH HETEROLOGOUS ANTISERA

G. P. ROBERTS

*From the University Department of Surgery, Welsh National School of Medicine, Heath Park, Cardiff*

Received 5 March 1980 Accepted 27 May 1980

**Summary.—Antisera were raised in rabbits to 6 human melanoma cell lines. The cell-surface antigens recognized by these antisera were examined using cell-surface labelling with $^{125}I$, followed by immunoprecipitation of soluble extracts of the cells and polyacrylamide-gel electrophoresis of the immunoprecipitates in the presence of sodium dodecyl sulphate (SDS). Up to 16 cell-surface antigens were recognized by these antisera, and 5 of the melanoma cell lines had a similar profile of cell-surface antigens. Digestion of labelled melanoma cells with neuraminidase before immunoprecipitation revealed that 8 of the larger antigens were sialoglycoproteins. The melanoma antisera produced haemagglutination of human erythrocytes at high dilutions, but the antigens involved could not be detected by iodination. In contrast, absorption of the melanoma sera with lymphocytes and fibroblasts revealed that these cells did contain some cell-surface glycoproteins in common with melanoma cells. The melanoma antisera contained antibodies to foetal calf serum proteins, but the amounts of these proteins on the surface of melanoma-cells were very low. Immunoprecipitation of labelled melanoma cell extracts with monospecific antiserum to $\beta_2$-microglobulin produced 2 bands with mol. wts corresponding to $\beta_2$-microglobulin and the HLA-determinant polypeptide chain. After absorption of melanoma antisera with cross-linked foetal calf serum, erythrocytes, lymphocytes and fibroblasts, antibodies against 10 labelled antigens remained in the absorbed antisera. However, antibodies against 8 of these antigens were still detected after absorption of the melanoma antisera with melanoma cells.**

THE CELL SURFACE of neoplastic cells is involved in many of the important behavioural traits which distinguish these cells from normal cells (Nicolson, 1976; Wallach, 1976). Changes in the cell-surface proteins occur on malignant transformation of cells *in vitro* with chemical carcinogens or viruses (Hynes, 1976). There are also many reports on the presence of tumour-associated antigens on human neoplasms, but these antigens have not been well characterized. In order to exploit the surface properties of human neoplastic cells for immunodiagnostic and immunotherapeutic purposes, more information is required about the surface antigens on these cells and their relationship to the surface components of normal cells.

In a previous study from this laboratory lactoperoxidase-catalysed iodination was used to label cell-surface proteins on human melanoma cells, which were then examined by polyacrylamide gel electrophoresis and detected by autoradiography (Roberts, 1978). In the present study antisera have been raised in rabbits against human melanoma cell lines and the cell-surface antigens recognized by these antisera have been characterized by immunoprecipitation of extracts of labelled melanoma cells.

### MATERIALS AND METHODS

*Cells.*—Fibroblast cultures were developed from operative biopsy specimens and cultured as described by Whitehead (1976). Two of the melanoma cell cultures (HTC 163 and HTC 364) were obtained from Dr R. H. Whitehead and have been described previously (Roberts, 1978). Four other melanoma cell cultures (Mel 2a, Mel 57, NK1-4 and RPM1-5966) were obtained from Dr M. J. Embleton. All the cells were cultured in McCoy's 5A containing the additives described previously (Roberts, 1978).

*Staphylococcus aureus* (Strain Cowan 1) was cultured and prepared for use in immunoprecipitation experiments as described by Kessler (1975).

*Antisera.*—Melanoma cells were cultured to confluency in roller bottles and then removed from the flasks by scraping with a rubber policeman. After centrifugation and removal of the supernatant the cell pellet was washed ×3 with phosphate-buffered saline (PBS), pH 7·2. The cells (10–14 × $10^6$ cells in total) were suspended in saline (0·7 ml) and mixed with Freund's complete adjuvant (0·7 ml). This mixture was injected s.c. into 4 sites on the back of a 2·5kg New Zealand white rabbit. Two further injections of melanoma cells (10–14 × $10^6$ cells) mixed with Freund's incomplete adjuvant were made at intervals of 14 days and then further injections were made at monthly intervals. Blood was taken from the rabbit's ear vein before immunization, on Day 36, and then monthly. Sera were heat-inactivated for 30 min at 56°C and stored in small aliquots at −20°C.

Antiserum to foetal calf serum was produced by a similar immunization schedule using a mixture of foetal calf serum (0·1 ml) (Flow Laboratories Ltd, Victoria Park, Irvine, Scotland), saline (0·3 ml) and Freund's complete adjuvant (0·4 ml) for the first injection and a similar mixture with Freund's incomplete adjuvant substituted for the complete adjuvant in subsequent injections.

Antiserum to bovine serum albumin was produced as described previously (Roberts & Parker, 1974). Antisera to tubulin and actin were generously provided by Dr R. A. Badley, who produced them as described previously (Badley *et al.*, 1978).

Antiserum to $\beta_2$-microglobulin was purchased from DAKO-Immunoglobulins, Copenhagen, Denmark.

*Absorption of antisera.*—An insoluble immunoadsorbent containing cross-linked foetal calf serum was prepared according to the method described by Fuchs & Sela (1973). Human blood from subjects in blood group A, B or O(H) was collected by venipuncture and placed in heparinized tubes. Lymphocytes were separated from erythrocytes by the method of Böyum (1968) and both the lymphocytes and erythrocytes were then washed several times with saline.

Antisera were absorbed with an equal volume of erythrocytes, lymphocytes, fibroblasts, melanoma cells or foetal calf serum immunoadsorbent at 37°C for 1 h and then at 4°C for 1 h. After centrifugation at 12,000 *g* for 5 min the supernatant was subjected to 2 further adsorptions under similar conditions to the first adsorption.

*Iodination.*—Cells were iodinated as described by Roberts (1978). Foetal calf serum was iodinated using chloramine-T according to the method described by Hunter (1973).

*Electrophoresis and autoradiography.*—SDS-gel electrophoresis was carried out on 5–22·5% gradient gels and the labelled antigens detected by autoradiography (ARG) as described previously (Roberts, 1978). Protein standards for mol.-wt estimations were myosin (200,000), $\beta$-galactosidase (130,000), phosphorylase a (94,000), bovine serum albumin (69,000), ovalbumin (45,000), myoglobin (17,000) and lysozyme (14,400).

*Immunoprecipitation.*—Labelled cells (1–2 × $10^6$) were extracted with 200 $\mu$l of 2% Nonidet P40 in 0·015M tris-HCl buffer (pH 7·5) containing 0·14M NaCl, 1·5mM magnesium chloride and 2mM phenylmethyl sulphonyl fluoride (PMSF) for 2 h at 4°C. After centrifugation at 12,000 *g* for 10 min aliquots (20 $\mu$l) of the supernatant were incubated with antisera (10 $\mu$l) for 1 h at 37°C and then for 1 h at 4°C. After this, 10% Nonidet P40 in PBS (20 $\mu$l) was added followed by 100 $\mu$l of 10% *Staphylococcus aureus* (Strain Cowan 1) in PBS and well mixed. The mixture was allowed to stand at 20°C for 1 h and then centrifuged for 1 min at 12,000 *g*. The cell pellet was washed ×4 with 2% Nonidet P40 in PBS (500 $\mu$l), care being taken to bring the cell pellet into an even suspension at each washing. Finally the cell pellet was suspended in 60 $\mu$l of 0·05M tris-HCl buffer (pH 6·8) containing 2% SDS, 1% mercaptoethanol, 10% glycerol, 0·001% bromophenol blue and 2mM PMSF, and heated for 3 min at 100°C. After centrifugation at 12,000 *g* for 3 min, aliquots (20 $\mu$l) of the supernatant were removed for electrophoresis.

*Neuraminidase treatment.*—Labelled cells (1–2 × $10^6$) were digested with 0·4 u of neuraminidase (Sigma Type V: purified from *Cl. perfringens*) in PBS, pH 7·4 (80 $\mu$l) containing 2mM phenylmethyl sulphonyl fluoride at 37°C for 1 h. Control cells were incubated in PBS under similar conditions, without the addition of neuraminidase. After incubation the cells were centrifuged at 12,000 *g* for 3 min and the supernatant discarded. The cell pellet was extracted with Nonidet P40 and the extracts used for immunoprecipitation experiments as described above.

*Haemagglutination.*—Serial dilutions of antisera were tested for their ability to agglutinate human erythrocytes using the method described by Hudson & Hay (1976).

### RESULTS

### *Surface antigens on melanoma cell line HTC 364 recognized by an antiserum raised against this cell line*

An autoradiograph of the electrophoretic pattern of the cell surface proteins extracted from melanoma cell line HTC 364 with Nonidet P40 is shown in Fig. 1. Twenty-one labelled bands were detected and the mol. wts are shown in the Table. The mol. wts quoted in this communication were all determined by comparison with the migration of mol.-wt markers on SDS gel electrophoresis. Consequently these are only approximate values, since it is known that the mol. wts of glycoproteins are over-estimated by this procedure (Pitt-Rivers & Impiombato, 1968). Immunoprecipitation of Nonidet P40 extracts of iodinated HTC 364 cells with an antiserum raised in a rabbit against this cell line, followed by recovery of the immune complexes with *Staphylococcus aureus* (Strain Cowan 1) (Kessler, 1975; Cullen & Schwartz, 1976) and subsequent electrophoresis and autoradiography, produced the pattern shown in Lane a of Fig. 1. Sixteen of the cell-surface components in the Nonidet P40 extracts were detected using this antiserum. A few of these bands were rather faint and although clearly detectable on the original autoradiograph they are difficult to detect in the reproduction in Fig. 1. When pre-immune serum from the same rabbit was substituted for the immune serum in the immunoprecipitation reaction, no bands were detected under the same conditions of electrophoresis and autoradiography. Comparison of the relative intensities of the bands in the Nonidet P40 extracts (Lane b, Fig. 1) and the immunoprecipitate (Lane a, Fig. 1) revealed that there are some variations. This may be related to differences in the titres of antibodies

FIG. 1.—ARG of $^{125}$I-labelled melanoma cell culture HTC 364 after electrophoresis on 5–22·5% polyacrylamide gels with SDS. (a) Immunoprecipitate of Nonidet P40 extracts of cells with antiserum to HTC 364; (b) Nonidet P40 extract of cells.

TABLE.—*Radioactive bands detected by ARG of SDS electrophoretic gels of melanoma cell line HTC* 364 *labelled with* $^{125}I$

| Mol. wts of bands $\times 10^{-3}$ | | | Intensity of bands in immuno-precipitate of untreated cells* |
|---|---|---|---|
| NI extract | Immuno-precipitate of untreated cells | Immuno-precipitate of neuraminidase-treated cells | |
| 233·5 | 233·5 | 218·8 | trace |
| 213 | 213 | 197·2 | + + + + |
| 194·1 | 194·1 | 184·1 | + |
| 173·7 | 173·7 | 166·0 | + + + + + |
| 154·1 | 154·1 | 142·2 | + + + |
| 135·5 | 135·5 | 124·5 | + + + + |
| 123·2 | 123·2 | 112·2 | + + + + |
| 112·5 | 112·5 | | trace |
| 104·1 | 104·1 | 104·1 | + + |
| 97·9 | 97·9 | 94·4 | + + + + + |
| 82·9 | 82·9 | 82·9 | + + |
| 77·2 | 77·2 | 77·2 | + + + |
| 74·6 | 74·6 | 74·6 | + + + |
| 50·1 | 50·1 | 50·1 | + + + + |
| 48·0 | 48·0 | 48·0 | + + + + |
| 34·9 | — | — | |
| 30·8 | — | — | |
| 30·4–27 | — | — | |
| 21·0 | — | — | |
| 19·3 | — | — | |
| 14·6 | 14·6 | 14·6 | |

* Assessed on a scale from trace to + + + + +.

against the different cell-surface components.

*Comparison of the cell-surface antigens on different melanoma cell lines*

The labelled cell-surface antigens detected in Nonidet P40 extracts of cell line NK1-4 by immunoprecipitation, using antisera raised against 5 different melanoma cell lines, are shown in Fig. 2. Immunoprecipitation with 3 of the antisera (raised against cell lines Mel 2a, HTC 364 and Mel 57) produced virtually identical electrophoretic patterns (Lanes b, c and e of Fig. 2). Antisera raised against cell line HTC 163 produced a somewhat different pattern, the bands of mol. wt 213,000, 50,100 and 48,000 were not detected and only small amounts of bands with mol. wts 82,900, 77,200 and 74,600 were detected. Surprisingly, the antiserum raised against cell line NK1-4 produced poor immunoprecipitation of 2 of the major bands, with mol. wts of 154,100 and 97,900, but the intensities of the other bands were similar to those produced with antisera against cell lines Mel 2a, HTC 364 and Mel 57. This suggests that the rabbit used to raise the antiserum against NK1-4 was a poor responder for these 2 antigens.

Immunoprecipitation of the labelled cell-surface antigens in Nonidet P40 extracts of 4 different melanoma cell lines (NK1-4, Mel 2a, RPMI-5966 and Mel 57) with antiserum to Mel 57 produced very similar electrophoretic patterns. With Mel 2a and RPMI-5966 there were no apparent differences between the cell-surface antigens recognized with their own antisera and with antisera raised against Mel 57.

These results show that most of the melanoma cell lines examined contain a

FIG. 2. — Immunoprecipitation of $^{125}I$-labelled melanoma cell culture NK1–4 with (a) antiserum to NK1–4, (b) antiserum to Mel 2a, (c) antiserum to HTC 364, (d) antiserum to HTC 163 and e) antiserum to Mel 57. Proteins were separated on 5–22·5% polyacrylamide gels in SDS and detected by ARG.

similar profile of cell-surface proteins that are immunogenic in the rabbit.

*Effect of neuraminidase on cell-surface proteins of melanoma cells*

Melanoma cell cultures were labelled with $^{125}I$ and then aliquots of these cells were treated with neuraminidase. Other aliquots of cells were incubated under similar conditions but without addition of neuraminidase. The cells were then extracted with Nonidet P40 and the extracts subjected to immunoprecipitation with their own antisera. An autoradiograph of the electrophoretic patterns of the immunoprecipitates is shown in Fig. 3. The electrophoretic migration of 7 cell-surface proteins in the mol.-wt range 112,500–233,500 and one protein with mol. wt 104,100 were all reduced by neuraminidase digestion, demonstrating that they are sialoglycoproteins. The migration of cell-surface proteins in the mol.-wt range 14,600–97,900 and one protein of mol. wt 112,500 was unchanged by neuraminidase digestion, indicating that they are proteins or glycoproteins lacking neuraminidase-susceptible sialic acid residues.

FIG. 3.—Effect of neuraminidase on the cell-surface antigens of melanoma cultures labelled with $^{125}I$. The lanes contained immunoprecipitates of (a) Mel 2a with antiserum to Mel 2a, (b) neuraminidase-treated Mel 2a with antiserum to Mel 2a, (c) RPM1-5966 with antiserum to RPM1-5966, (d) neuraminidase-treated RPM1-5966 with antiserum to RPM1-5966, (e) Mel 57 with antiserum to Mel 57, (f) neuraminidase-treated Mel 57 with antiserum to Mel 57, (g) HTC 364 with antiserum to HTC 364 and (h) neuraminidase-treated HTC 364 with antiserum to HTC 364. Proteins were separated on 5–22·5% polyacrylamide gels with SDS and detected by ARG.

*Absorption of antisera*

Antisera raised against melanoma cell lines HTC 364, Mel 2a, Mel 57, RPMI-5966 and HTC 163 all caused haemagglutination of human Group O(H) red blood cells down to dilutions of 1:1280. Antiserum against melanoma cell line NK1-4 produced haemagglutination down to a dilution of 1:320. None of the preimmune sera produced haemagglutination at any of the dilutions tested (1:5 to 1:10240). Absorption of the antisera 3 times with an equal volume of packed red blood cells removed this haemagglutination activity completely. However, comparison of the labelled cell-surface proteins precipitated by the absorbed antisera with those precipitated by the original antisera revealed no difference. This suggests that the red-blood-cell antigen recognized by the original antisera is not a protein labelled by the iodination. Absorption of the melanoma antisera with glutaraldehyde-cross-linked foetal calf serum produced very little change in the electrophoretic patterns of cell-surface proteins in the immunoprecipitates, when compared with immunoprecipitates detected with the unabsorbed antisera. However, immunoprecipitation of labelled malanoma cell extracts with antiserum raised against foetal calf serum did reveal trace amounts of proteins originating from FCS on the melanoma cell surface. In the electrophoretic patterns obtained from immunoprecipitates produced with the melanoma antisera, these trace components are obscured by more intense bands produced by integral melanoma surface components. However, the trace amounts of foetal calf serum on the melanoma cell surface are sufficient to elicit antibody formation in rabbits, and the melanoma antisera do contain considerable amounts of antibodies directed against FCS components. This was demonstrated by immunoprecipitation of $^{125}$I-labelled foetal calf serum with melanoma antisera, when precipitation of most of the components of labelled foetal calf serum occurred. Absorption of antiserum to HTC 364 with lymphocytes before immunoprecipitation of labelled HTC 364 extracts produced a large decrease of 2 of the major antigens with mol. wts of 173,700 and 154,100 in the electrophoretic pattern (Fig. 4, Lane c). Similarly absorption of antiserum to HTC 364 with adult fibroblasts caused a large decrease in 4 labelled bands with mol. wts of 173,700, 154,100, 135,500 and 50,100 (Fig. 4, Lane d). Confirmation that most of these antigens were common to fibroblasts and melanoma cells was obtained by labelling adult fibroblasts with $^{125}$I, and carrying out immunoprecipitation experiments with Nonidet P40 extracts of these cells and antiserum directed against HTC 364. Autoradiographs of electrophoretic patterns of the immunoprecipitate revealed 4 bands with mol. wts of 173,700, 154,100, 135,000 and 112,500 (Fig. 4, Lane e). Absorption of melanoma antisera with melanoma cells of the line used to raise the antisera did not remove all the antibodies to cell-surface components. An autoradiograph of the electrophoretic pattern obtained from an immunoprecipitate of melanoma cell line HTC 364 with melanoma-cell-absorbed antisera revealed 8 persistent antigens with mol. wts of 233,500, 123,000, 104,100, 97,900, 82,900, 77,200, 74,600 and 48,000 (Fig. 4, Lane a). The antibodies to these cell-surface components survived 3 absorptions of antisera with an equal volume of packed tumour cells in each absorption. Similar immunoprecipitation experiments with melanoma cell line Mel 57 revealed that antibodies against 6 antigens remained after absorption of antisera raised against this line with Mel 57 cells. An identical pattern was obtained when HTC 364 cells were used to absorb the antiserum raised against cell line Mel 57.

FIG. 4.—Effect of absorption of antisera before immunoprecipitation of cell-surface antigens. Immunoprecipitation experiments were carried out with $^{125}$I-labelled melanoma cell culture HTC 364 and antiserum to HTC 364 absorbed with (a) HTC 364 cells, (b) cross-linked foetal calf serum and erythrocytes, (c) human lymphocytes and (d) adult human fibroblasts with unabsorbed antisera to HTC 364. Proteins were separated on 5–22·5% polyacrylamide gels with SDS and detected by ARG.

*Immunoprecipitation of melanoma cell-surface components with mono-specific antisera*

A faint band of albumin was detected in immunoprecipitates produced by reaction of extracts from labelled HTC 364 with antiserum to bovine serum albumin. Incubation with antiserum to $\beta_2$-microglobulin resulted in precipitation of 2 antigens which were assigned mol. wts of 48,000 and 14,600. Immunoprecipitation with antisera directed against actin and tubulin produced no radioactive bands.

### DISCUSSION

The detection of cell-surface components by surface labelling followed by SDS electrophoresis and autoradiography has proved to be a powerful technique for studying the externally orientated proteins and glycoproteins of the plasma membrane (Hubbard & Cohn, 1975). Combination of this technique with immunoprecipitation using specific antisera and isolation of the immunoprecipitates with protein A-bearing staphylococci has allowed the successful characterization of surface antigens such as surface immunoglobulins (Kessler, 1975) and histocompatibility antigens (Cullen & Schwartz, 1976). In the present investigation this technique has been used to examine the surface antigens on human melanoma cells. Most of the cell-surface components with mol. wts $> 49{,}000$ were antigenic in the rabbit; 21 bands were detected in the Nonidet P40 extracts and 16 in the immunoprecipitates (Fig. 1). Recent work using lectin affinity chromatography has shown that some labelled bands in the Nonidet P40 extracts may be masked in the electrophoretic pattern by other more intense bands (Roberts, unpublished). Consequently these figures for the number of labelled bands may be underestimates. One feature of the present investigation is that a number of similar antigens are precipitated from a single cell line by antisera raised against different cell lines (Fig. 2). This indicates that the different melanoma cell lines have a number of antigens in common. Confirmation of this was obtained by immunoprecipitation experiments with extracts of different melanoma cells using a single antiserum. A previous study from this laboratory (Roberts, 1978) using iodination without immunoprecipitation revealed considerably more differences between 6 melanoma cell cultures at low passage levels. It was suggested that variations in degree of glycosylation of cell-surface glycoproteins might explain the differences in electrophoretic patterns of cell-surface components from different melanoma cultures. The results presented here do show that at least 8 of the labelled bands contain sialic acid, removal of which changes their migration characteristics. Surface labelling of melanoma cells with galactose oxidase/Na $B^3H_4$ has revealed that the surface glycoproteins were not labelled unless sialic acid was first removed with neuraminidase (Lloyd *et al.*, 1979; Roberts, unpublished).

The sialic acid content of tumours has aroused much interest (Van Beek *et al.*, 1973). The glycoproteins of virus-transformed cells have been shown to be more highly sialylated than the glycoproteins of untransformed cells (Warren *et al.*, 1975). In studies with low and high lung-metastasizing variants, Yoogeeswaran *et al.* (1978) showed that the highly tumorigenic and metastatic mouse melanoma cells were enriched for highly sialylated glycoproteins. A high degree of sialylation of surface antigens might aid a highly invasive and metastatic tumour such as a human melanoma to evade destruction by the host's immune response, if the sialic acid represses underlying antigenic determinants as has been suggested previously (Apffel & Peters, 1970; Quish & Lange, 1973).

Monolayer cell cultures have been reported to bind serum proteins (Phillips & Perdue, 1977). This is confirmed in the present investigation, which has shown that antisera produced against cultured melanoma cells contain antibodies reacting with FCS proteins. However, the amounts of FCS proteins absorbed by the melanoma cells are very low, and are not readily detected in the immunoprecipitates formed between melanoma extracts and anti-melanoma sera, due to masking by more intense bands composed of integral membrane proteins.

Other workers (Barnstable *et al.*, 1978) have shown that $\beta_2$-microglobulin (mol. wt 12,000) is noncovalently associated on cell surfaces with a polypeptide (mol. wt 44,000) which carries the HLA antigenic specificity. Two bands assigned mol. wts of 48,000 and 14,600 were precipitated from Nonidet P40 extracts of iodinated melanoma cells with antiserum to $\beta_2$-microglobulin. The differences in the 2 sets of mol wts are within the accuracy expected for the method of mol. wt determination used. The expression of Ia-like antigens on melanoma cells has been described by 2 groups of investigators (Wilson *et al.*, 1979; Winchester *et al.*, 1978). However, in the present study, bands corresponding to the bimolecular glycoprotein with Ia-like activity were not detected in immunoprecipitates of melanoma extracts with anti-melanoma sera.

The antisera raised against melanoma cells caused haemagglutination of human erythrocytes at high dilutions, but the antigen(s) involved did not appear to be a protein or glycoprotein which could be labelled by iodination. Erythrocytes from donors of blood groups A, B and O(H) were agglutinated by similar dilutions of antisera, indicating that the antigen is not a glycolipid with ABO(H) activity. Absorption of the anti-melanoma sera with fibroblasts and lymphocytes did reveal that these cell types possessed some cell-surface antigens in common with melanoma cells. This was confirmed in the case of fibroblasts by labelling fibroblasts and reacting them with anti-melanoma sera. Antibodies against several antigens were not removed from anti-melanoma serum by absorption with melanoma cells. Possible explanations for the persistence of these antigens are that the antibodies against them are present in very large amounts, and that the absorption although extensive was not complete, or that the approach of immunoglobulin to these antigens is sterically hindered by the large glycoproteins surrounding them. In this connection it is of interest that Sanford *et al.* (1973) and Codington *et al.* (1973) have suggested that the lack of strain specificity in the mammary carcinoma ascites cell TA3-Ha is due to masking of cell-surface histocompatibility antigens by sialoglycoprotein molecules of high mol. wt which are present at the surface of these cells. These sialoglycoproteins are absent from the surface of strain-specific TA3-S1-ascites cells.

In addition to the antigens the antibodies of which were not removed from the anti-melanoma sera by absorption with melanoma cells, there were at least 2 antigens (mol. wts 213,000 and 194,100) the antibodies of which were removed by absorption of the anti-melanoma sera with melanoma cells but not with lymphocytes or fibroblasts. While it cannot be claimed at this stage that there are melanoma-associated antigens, it will be important in future studies to establish whether antibodies against them can be absorbed out of the antisera by any other cell type, and to determine the cellular specificity of monospecific antisera raised against these antigens.

I wish to thank Dr M. J. Embleton and Dr R. H. Whitehead for supplying the cell cultures, Miss Gwenda Roberts, Mr D. L. Jones and Mr J. Thatcher for technical assistance and Professor L. E. Hughes for advice.

This study was supported by a grant from the Cancer Research Campaign.

### REFERENCES

Apffel, C. A. & Peters, J. H. (1970) Regulation of antigenic expression. *J. Theoret. Biol.*, **26**, 47.

Badley, R. A., Lloyd, C. W., Woods, A., Carruthers, L., Allcock, C. & Rees, D. A. (1978) Mechanisms of cellular adhesion. III Preparation and preliminary characterisations of adhesions. *Exp. Cell. Res.*, **117**, 231.

Barnstable, C. J., Jones, E. A. & Crumpton, M. J. (1978) Isolation, structure and genetics of HLA-A, -B, -C and -DRW (Ia) antigens. *Br. Med. Bull.*, **34**, 241.

Böyum, A. (1968) Isolation of mononuclear cells and granulocytes from human blood. *Scand. J. Clin. Lab. Invest.*, **21**, 77.

Codington, J. F., Sandford, B. H. & Jeanloz, R. W. (1973) Cell-surface glycoproteins of two sublines of the TA3 tumor. *J. Natl Cancer Inst.*, **51**, 585.

Cullen, S. E. & Schwartz, B. D. (1976) An improved method for isolation of H-2 and Ia alloantigens with immunoprecipitation induced by protein A-bearing staphylococci. *J. Immunol.*, **117**, 136.

Fuchs, S. & Sela, M. (1973) Immunoadsorbents. In *Handbook of Experimental Immunology. Vol. I Immunochemistry.* Ed. Weir. Oxford: Blackwell Sci. Publ. p. 11.

Hubbard, A. L. & Cohn, Z. A. (1975) Externally disposed plasma membrane proteins I. Enzymatic iodination of mouse L cells. *J. Cell. Biol.*, **64**, 438.

Hudson, L. & Hay, F. C. (1976) *Practical Immunology.* Oxford: Blackwell Sci. Publ. p. 125.

HUNTER, W. M. (1973) Radioimmunoassay. In *Handbook of Experimental Immunology Vol. 1 Immunochemistry*. Ed. Weir. Oxford: Blackwell Sci. Publ. p. 17.

HYNES, R. O. (1976) Cell surface proteins and malignant transformation. *Biochim. Biophys. Acta*, **458**, 73.

KESSLER, S. W. (1975) Rapid isolation of antigens from cells with a staphylococcal protein A-antibody adsorbent: Parameters of the interaction of antibody–antigen complexes with Protein A. *J. Immunol.*, **115**, 1617.

LLOYD, K. O., TRAVASSOS, L. R., TAKAHASHI, T. & OLD, L. J. (1979) Cell surface glycoproteins of human tumor cell lines: Unusual characteristics of malignant melanoma. *J. Natl Cancer Inst.*, **63**, 623.

NICOLSON, G. L. (1976) Transmembrane control of the receptors on normal and tumor cells. II Surface changes associated with transformation and malignancy. *Biochim. Biophys. Acta*, **458**, 1.

NILSSON, K., ANDERSSON, L. C., GAHMBERG, C. G. & WIGZELL, H. (1977) Surface glycoprotein patterns of normal and malignant lymphoid cells. II. B cells, B blasts and Epstein–Barr virus (EBV)-positive and -negative B lymphoid cell lines. *Int. J. Cancer*, **20**, 708.

PITT-RIVERS, R. & IMPIOMBATO, F. S. A. (1968) The binding of sodium dodecyl sulphate to various proteins. *Biochem. J.*, **109**, 825.

PHILLIPS, E. R. & PERDUE, J. F. (1977) Immunologic identification of fetal calf serum-derived proteins on the surfaces of cultured transformed and untransformed rat cells. *Int. J. Cancer*, **20**, 798.

QUISH, T. B. & LANGE, C. F. (1973) Increased antigenicity of glycoproteins after carbohydrase treatment. *Res. Commun. Chem. Pathol. Pharmacol.*, **5**, 473.

ROBERTS, G. P. (1978) Lactoperoxidase-catalysed iodination of surface proteins on human melanoma cells. *Br. J. Cancer*, **38**, 114.

ROBERTS, G. P. & PARKER, J. M. (1974) Macromolecular components of the luminal fluid from the bovine uterus. *J. Reprod. Fert.*, **40**, 291.

SANFORD, B. H., CODINGTON, J. F., JEANLOZ, R. W. & PALMER, P. D. (1973) Transplantability and antigenicity of two sublines of the TA3 tumor. *J. Immunol.*, **110**, 1233.

VAN BEEK, W. P., SMETS, L. A. & EMMELOT, P. (1973) Increased sialic acid density in surface glycoprotein of transformed and malignant cells—A general phenomenon? *Cancer Res.*, **33**, 2913.

WALLACH, D. F. H. (1976) Membrane anomalies of neoplastic cells. *Med. Hypoth.*, **2**, 241.

WARREN, L., FUHRER, J. P. & BUCK, C. A. (1972) Surface glycoproteins of normal and transformed cells: A difference determined by sialic acid and growth dependent sialyl transferase. *Proc. Natl Acad. Sci. U.S.A.*, **68**, 1838.

WARREN, L., ZEIDMAN, I. & BUCK, C. A. (1975) The surface glycoproteins of a mouse melanoma growing in culture and as a solid tumor *in vivo*. *Cancer Res.*, **35**, 2186.

WILSON, B. S., INDIVERI, F., PELLEGRINO, M. A. & FERRONE, S. (1979) DR (Ia-like) antigens on human melanoma cells. Serological detection and immunochemical characterisation. *J. Exp. Med.*, **149**, 658.

WINCHESTER, R. J., WANG, C.-Y., GIBOFSKY, A., KUNKEL, H. G., LLOYD, K. O. & OLD, L. J. (1978) Expression of Ia-like antigens on cultured human malignant melanoma cell lines. *Proc. Natl Acad. Sci. U.S.A.*, **75**, 6235.

WHITEHEAD, R. H. (1976) The culture of tumour cells from human tumour biopsies. *Clin. Oncol.*, **2**, 131.

YOOGEESWARAN, G., STEIN, B. S. & SEBASTIAN, H. (1978) Altered cell surface organization of gangliosides and sialylglycoproteins of mouse metastatic melanoma variant lines selected *in vivo* for enhanced lung implantation. *Cancer Res.*, **38**, 241.